# Social and Structural Determinants of Health and Social Injustices Contributing to Obesity Disparities

**DOI:** 10.1007/s13679-024-00578-9

**Published:** 2024-06-15

**Authors:** Michelle S. Williams, Sheila J. McKinney, Lawrence J. Cheskin

**Affiliations:** 1 George Mason University, College of Public Health, Department of Global and Community Health, Fairfax, VA 22030 United States; 2 Jackson State University, School of Public Health, Department of Epidemiology and Biostatistics, Jackson, MS 39217 United States; 3 George Mason University, College of Public Health, Department of Nutrition and Food Studies, Fairfax, VA 22030 United States; 4 Johns Hopkins University School of Medicine, Department of Medicine, 1830 E. Monument Street, Baltimore, MD 21205 USA

**Keywords:** Obesity, Race, Ethnicity, Health disparities, Socioeconomic status, Social determinants of health, Structural determinants of health, Social injustice

## Abstract

**Purpose of Review:**

To analyze how social and structural determinants of health and social injustice impact the risk of obesity, its treatment and treatment outcomes, and to explore the implications for prevention and future treatment interventions.

**Recent Findings:**

Racial and ethnic minorities, such as non-Hispanic Black adults and Hispanic adults, and adults with a low socioeconomic status have a greater risk of obesity than non-Hispanic white adults and adults with a high socioeconomic status. The underlying causes of obesity disparities include obesogenic neighborhood environments, inequities in access to obesity treatment, and lack of access to affordable nutrient-dense foods. Experts have called for interventions that address the social and structural determinants of obesity disparities. Population-based interventions that focus on improving neighborhood conditions, discouraging the consumption of unhealthy foods and beverages, expanding access to obesity treatment, and ensuring equitable access to fruits and vegetables have been proven to be effective.

**Summary:**

There is a growing body of evidence that shows the relationship between social and structural determinants of health and injustice on disparities in obesity among racial and ethnic minorities and individuals with a low SES. Population-based, equity-focused interventions that address the underlying causes of obesity disparities are needed to reduce obesity disparities and improve the health outcomes of minoritized and marginalized groups.

## Introduction

Obesity is a major public health issue in the US. Cardiovascular disease, many forms of cancer, and type-2 diabetes are obesity-related conditions that contribute to the continual decline in life expectancy [1]. In addition, obesity was one of the comorbidities associated with increased risk of mortality due to COVID-19 between 2020 and 2022 [2–4]. The prevalence of obesity has been steadily increasing among adults of all races and ethnicities over the last four decades [5, 6]. However, the prevalence of obesity is significantly higher among Non-Hispanic Black adults, Hispanic adults, and American Indian or Alaska Native adults than their White and Asian counterparts [7–9]. Non-Hispanic Black women have the highest prevalence of obesity (57.9%) and severe obesity (19.1%) among all adults in the US [9]. Among men, Non-Hispanic Black men have the highest prevalence of severe obesity (7.9%), and Hispanic men have the highest prevalence of obesity (45.2%) [5].

**Fig. 1:**
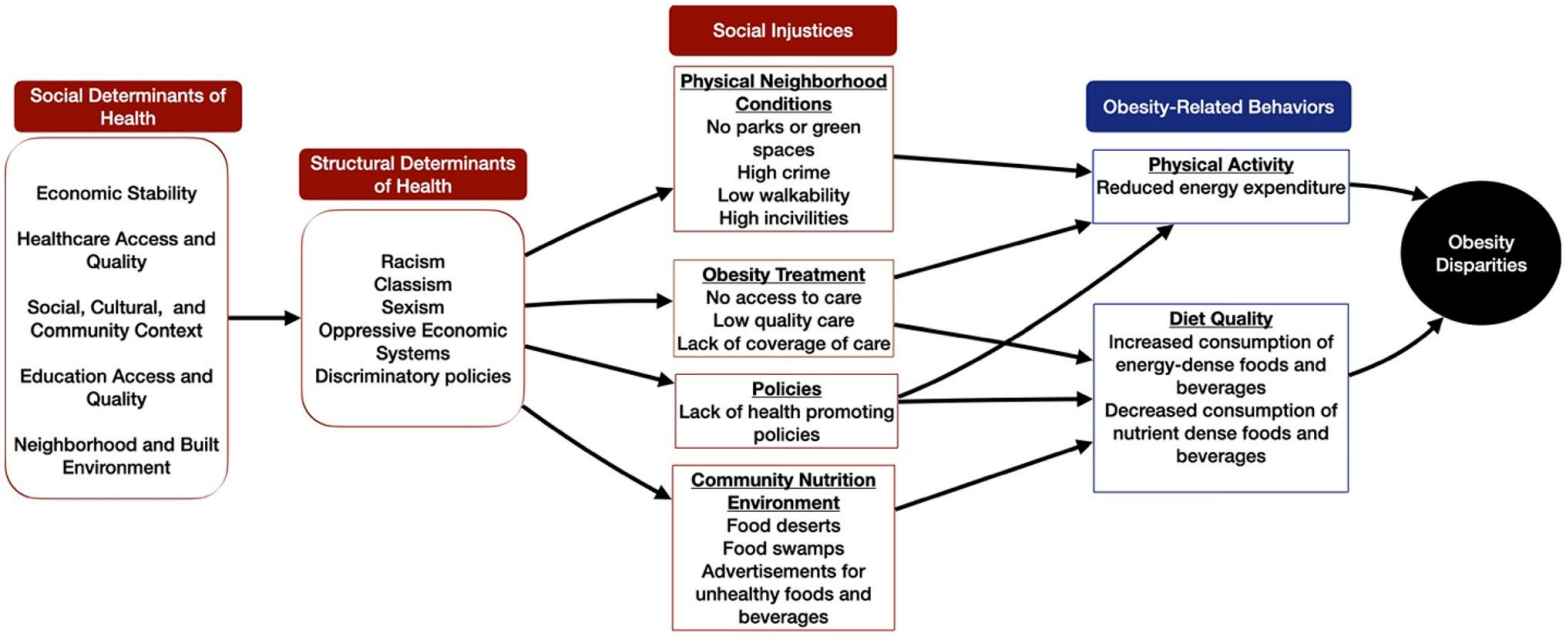
Social and structural determinants of health and social injusticies that contribute to obesity disparities

Evidence suggests that social determinants of health (SODH), structural determinants of health, and social injustices are the root causes of modifiable factors associated with disparities in the prevalence of obesity among minoritized populations [10–13]. According to the US Department of Health and Human Services, the SODH are “the conditions in the environments where people are born, live, learn, work, play, worship, and age that affect a wide range of health, functioning, and quality-of-life outcomes and risks” [14]. The overarching SODH domains include the neighborhood and built environment, healthcare access and quality, economic stability, education access and quality and social and community context [14]. Structural determinants of health are factors such as policies, practices, and institutions that influence the way people are affected by SODH [15]. For example, because of unjust housing practices, such as redlining, racial and ethnic minorities are more likely to live in disadvantaged neighborhoods that do not provide adequate access to quality healthcare, education, or a health-enhancing built environment [16–19]. As a result, individuals living in disadvantaged neighborhoods experience a higher SODH burden and subsequently a higher risk of obesity [20, 21]. Social injustices occur when there are inequities in the allocation of resources, opportunities and support for human rights based on one’s “disability, ethnicity, gender, age, sexual orientation or religion” [22]. The prevalence of food deserts, food swamps, and physical activity deserts in predominately Black, Hispanic, and low-income neighborhoods are examples of social injustices that limit access to core the elements of healthy living [22–24]. The relationship between the social and structural determinants of health, social injustices, and obesity disparities is depicted in Fig. 1.

Obesity interventions that address the specific social and structural determinants of health and social injustices that impact racial and ethnic minorities have been shown to be effective at reducing the prevalence and incidence of obesity, and improving access to treatment compared to individual level interventions [21, 25–28]. The purpose of this review is to elucidate the root causes of disparities in obesity among racial and ethnic minority adults in the US and to provide examples of actionable, evidence-based interventions to reduce obesity disparities.

## Methods

A systematic literature review was conducted to identify current evidence regarding the influence of social and structural determinants of health and social injustice on obesity disparities among racial and ethnic minorities. We searched databases, including PubMed and EBSCOhost to identify sources that were published between 2018 and 2023. Articles that were written in English, and focused on obesity disparities among racial and ethnic minorities in the US were retained.

## Results

### Social Inequities and Social Injustices that Contribute to Disparities in Obesity

The disparities in obesity among Black and Hispanic individuals can be attributed to several population-level factors, including differences in access to healthful foods, access to safe places to be physically active, access to high quality weight management and obesity treatment services, and health policies related to obesity [28]. Social inequities and social injustices are the underlying causes of many of the social and structural determinants of health that contribute to obesity disparities [11, 20, 21, 27, 29–31]. For example, the results of a study using data from the Behavioral Risk Factor Surveillance System showed that regardless of race and ethnicity, residents of low-income communities with food and physical activity deserts have higher BMI levels than residents of high income communities that have access to active commuting and fewer unhealthy food outlets [31]. In addition, Graham et al. [32] found that veterans living in counties with fewer farmer’s markets and natural amenities were less likely to be engaged in the Veterans Health Administration MOVE! Program.

### Health Care Services

Despite the existence of increasingly effective clinical treatments for obesity, evidence shows that Black and Hispanic individuals, particularly those of lower socioeconomic status, are less likely to be engaged in clinical obesity treatment or experience positive outcomes after receiving clinical obesity treatment [33, 34]. Black and Hispanic individuals living in medically underserved areas also have less access to healthcare providers trained to provide obesity treatment and preventionservices [35, 36]. Solutions are needed to increase access to obesity treatment through primary care provider training, using a team-based care model or other strategies [37].

Other factors that may contribute to disparities in obesity treatment outcomes include the lack of health insurance and the lack of access to high-quality healthcare services [33, 36, 38]. Race also influences obesity outcomes, notably following bariatric surgery, where Caucasian patients have been shown to achieve greater percent loss of excess body weight than Black patients [39]. The strict requirements for preoperative supervised weight loss have also been found to be associated with higher attrition rates for Black patients who were seeking bariatric surgery [40, 41].

Obesity treatment among Black and Hispanic children is especially challenging due macro-level and micro-level factors that are impacted by social and structural determinants of health [42]. While there are effective treatments for childhood obesity, Black and Hispanic children tend to have poorer outcomes than their White counterparts [35, 43, 44]. Less access to healthful foods, fewer safe places to be physically active, unmet social needs, low parental socioeconomic status and differing social expectations regarding weight status are key factors that may drive the poorer treatment outcomes among Black and Hispanic children with obesity [12, 43, 45–48].

### Federal, State, Local and Institutional Policies

There are federal, state, and local public health policies that are associated with reducing obesity disparities. Leadership represents organizations, researchers, advocates, policymakers, and government entities with a common goal: to promote a society where all groups can live healthy lives and avoid the various ailments, diseases, morbidity, and mortality associated with obesity [49]. Across federal, state, and local jurisdictions, leaders create health policies that set the stage for health interventions. As an example, *Healthy People* serves as the prevention agenda guiding the development of all federal public health policies, and these policies, in turn, frame the interventions created to prevent, control, or eliminate population morbidity and mortality [49]. This agenda uses science-backed evidence to promote the various goals and objectives that are the foundation of all public health programming. The evidence shows that (1) social determinants of health impact the quality of life, well-being, and overall health of a population, and (2) upstream factors are more influential on population outcomes than individual-level factors [50]. Therefore, to impact obesity in the US substantially, social determinants and upstream factors are vital components for health policies to consider.

Some policies have effectively applied this information to generate practical solutions through legislation, regulations, actions, and decisions that promote wellness among adults. At the forefront of these policies is the idea of behavior modification, whereby the language of the policies included strategies that informed adults with the appropriate declarative information to support selecting healthful foods for consumption and directed the procedures or steps to execute the ideal behaviors in preparing, buying, and integrating food into a healthier dietary practice. The policies promoted in the US typically stratify their approach by the age of the population (adults versus youth) and the target setting (community versus work environments). Within these strata, two behaviors, diet, and exercise, are hailed as the ultimate change agents to reduce obesity rates within a target population or to promote equity across disproportionate rates of obesity. For example, the CDC has sponsored three state-level programs to address obesity in the US [51]. Sixteen states were awarded close to 1 million dollars each to support the implementation of evidence-based strategies that encourage healthful eating, increased physical activity, and breastfeeding under the *State Physical Activity and Nutrition* (SPAN) program [51].

Behavior modification has extended to other relevant areas, including increasing the proportion of women breastfeeding their infants through one year postpartum. The underlying objective of breastfeeding programs was to inform women about the benefits of breastfeeding and, through collaborations with medical facilities, offer new mothers procedural support for feeding their infants before leaving the hospital or other birthing center [52–55]. Policies were created within hospitals to hire lactation specialists; some facilities collaborate with doulas and have secured funding to provide manual pumps to new mothers upon discharge [52–55]. While directed at individuals, these programs have had inconsistent success because they depend on support or buy-in from institutions, workplace environments, educational settings, and community venues to achieve substantive change in targeted behaviors [52, 53].

### Obesity and Systemic Racism, Structural Racism

Emerging evidence reveals that systemic and structural racism has fostered social policies that limit access to essential services, racial and ethnic minorities [17]. In the 1930s, the Federal Homeowner’s Loan Corporation created maps of 239 US cities whose topography showed in red areas, hence ‘redlining’ those neighborhoods as economic risks for issuing home loans based solely on the racial composition of its residents [56]. Policies such as redlining sanction social, political, and economic marginalization and segregation of the residents and communities from more affluent areas [57]. These outcomes become rooted in the system, contribute to health disparities, and may persist even after policy corrections [56]. Structural racism drives obesity disparities through limiting opportunities, economic disadvantage, and the absence of quality healthcare services [57]. For example, the mental and physiological effects of frequent experiences of racism, such as racial microaggressions, have been associated with increased risk of obesity in Black adults [58, 59].

### Community Environments

The built environment consists of the artificial and potentially modifiable structures that provide people with living, working, and recreational spaces [19, 60]. The built environment of neighborhoods with predominately racial and ethnic minority residents often lack health promoting features, such as green spaces and full-service supermarkets or grocery stores [19, 60]. Such communities have been described as *food deserts*, due to their limited access to nutrient dense foods, or *food swamps*, due to the overabundance of low-cost fast food and junk food [61]. Obesity is also more prevalent in communities that do not provide safe environments for recreational activities [62, 63]. In addition, studies have found that minority and low-income neighborhoods are particular targets of marketing campaigns that promote foods and beverages high in fat and sugar [63]. Zoning laws that lead to restricted access to healthful foods, and the strategies used to promote the consumption of unhealthy foods are social injustices that contribute to obesogenic community environments [20, 21, 30, 64–66].

### Interventions to Reduce Racial/Ethnic Disparities in Obesity

Reducing obesity disparities among racial and ethnic minorities requires a multi-level approach [21, 37, 67]. Evidence-based interventions focused on institutional level, community level, and policy level factors have been shown to be effective at reducing the burden of obesity-related diseases in predominantly racial and ethnic minority communities. Table 1 provides examples of promising obesity interventions aimed at increasing equitable access to neighborhood conditions, health care services, and policies that promote healthy behaviors.

**Table 1:** Promising evidence-based interventions to reduce obesity disparities

**Intervention**	**Intervention Description**	**Key Strategies for Reducing Disparities**	Citation
**Neighborhood Conditions And Resources**			
Safe Environments for Physical Activity	The Parks After Dark program extended park operation hours during the summer months; Provided a safe environment for physical activity; Hosted programming to promote social cohesion and community well-being, such as health cooking demonstration	Community-level intervention that involved multiple sectors, including the County Department of Parks and Recreation, the Sheriff’s Department, and the Department of Public Health	[62]
Park Restoration	The Study of Active Neighborhoods in Detroit focused on restoring urban greenspaces	Community-level intervention focused on park restoration including replacing non-native plants and turf with native plants, creating trails, posting signage, and leading community stewardship events like bird watching walks	[63]
Civic Engagement	The Change Club is a civic engagement intervention targeted toward residents of rural communities	Interpersonal level and community-level intervention that was focused increasing collective efficacy and training residents to rural communities to make changes in their built environment that promote physical activity and healthy eating	[68]
**Local, State, and Federal Policies**			
Sugar Sweetened Beverage Tax	A one cent per ounce excise tax on sugar sweetened beverages	Policy-level intervention focused on reducing the consumption of sugar sweetened beverages which are easy to access in predominately racial and ethnic minority, and low-income neighborhoods	[70]
**Health Care Services**			
Expanding coverage of obesity treatment	Increasing the coverage of evidence-based obesity treatments by Medicaid and state employee health insurance programs	Policy-level intervention aimed at expanding coverage the coverage of bariatric surgery, nutrition counseling and pharmacotherapy for individuals Medicaid recipients and individual who use state employee health insurance program may increase equitable access to obesity treatment	[69]
**Community Nutrition Environments**			
Healthy Retail	The Tribal Health and Resilience in Vulnerable Environments (THRIVE) intervention focused on increasing access to fruits and vegetables in food deserts	Community-level intervention focused on increasing accessing to affordably priced fruits and vegetable in rural communities	[74]
Expanded acceptance of nutrition benefits programs	Increase the number of farmer’s markets that accept nutrition benefits programs	Community-level intervention focused on increasing access to healthy foods in food deserts	[61]

#### Neighborhood Conditions and Resources

Making neighborhoods safer for physical activity can be accomplished by improving lighting, building more parks and playgrounds, and making it easier for people to walk and bike. Evidence has also demonstrated that involving multiple sectors, such as the health departments, department of parks and recreation, and law enforcement agencies, can foster safe environments that promote physical activity and potentially prevent excessive weight gain. The *Parks After Dark* program that was implemented in Los Angeles County is an example of a community-level intervention that was effective at increasing engagement in physical activity among adults and youth in predominantly Hispanic and African American neighborhoods [62]. It is estimated that since it was first implemented, the *Parks After Dark* program resulted in an estimated reduction of disease burden of 11 additional years of life expectancy and 11 fewer years of disability [62]. The *Change Club* is an example of multi-level intervention, interpersonal-level and community level, aimed at reducing barriers to physical activity and healthy eating in rural communities [68].

#### Health Care Services

Improving equitable access to obesity treatment can be accomplished by expanding health insurance coverage among governmental and private insurers, providing more funding for community health centers, and training more healthcare providers, particularly providers drawn from underrepresented populations [20, 28, 34, 38, 47, 69, 70]. Many individuals without adequate health insurance are not able to afford the cost of evidence-based obesity treatments such as bariatric surgery [33, 39, 41]. Evidence suggests that expanding the coverage of obesity treatments for Medicaid recipients and participants in state employee health insurance programs would increase access to those services [71].

#### Federal, State, Local and Institutional Policies

Added sugar intake, including sugar-sweetened beverage (SSB), is associated with a greater risk of incident obesity among young adults [32]. Racial and ethnic minorities and individuals with low incomes have significantly higher consumption of SSBs which can be attributed to poor community nutrition environments [72]. Taxing SSBs is a strategy aimed at reducing the consumption of SSBs. For example, the Berkeley, California City Council implemented a penny-per-ounce excise tax on sugar-sweetened beverages in 2016 [72]. The increased cost resulted in a decreased consumption of SSBs, which could be helpful in preventing obesity [73]. The outcomes of microsimulation studies suggest that state-level SSB tax is a cost-effective strategy for improving SSB-related health equity [74, 75].

#### Community Nutrition Environment

Access to healthful food can be increased by reducing the prevalence of food deserts and food swamps in low-income communities, providing subsidies for healthy food, and reducing access to unhealthy foods [61, 70, 76–78]. For example, there was a significant increase in the purchase of fruits and vegetables in the convenience stores in which the Tribal Health and Resilience in Vulnerable Environments (THRIVE) intervention was outcomes among Black and Hispanic children withimplemented [78]. In addition, expanding the acceptance of nutrition benefits programs such as the Supplemental Nutrition Benefits Program, has been associated with an increase in fruits and vegetables among individuals living in food deserts [61].

## Conclusion

Substantial disparities in obesity persist among racial and ethnic minorities and individuals with a low income. The evidence is strong that modifiable social and structural determinants of health and social injustices are primary drivers of obesity disparities. Interventions at multiple levels of the Socioecological Model are necessary to definitively address these underlying factors. Current evidence has demonstrated that there are a number of effective strategies for addressing community-level and policy-level structural and social determinants of health. However, more work needs to be done to expand and broaden these interventions in the many communities that continue to be negatively impacted by social and structural determinants of health and social injustices.

## Data Availability

No datasets were generated or analysed during the current study.
